# Synthetic Phosphorylation Networks with Fluorescence and Luminescence Expansion

**DOI:** 10.1021/acssynbio.4c00814

**Published:** 2025-06-06

**Authors:** Leah Davis, Evan J. Hutt, Matthias Recktenwald, Samarth Patel, Madison Briggs, Madeline Dunsmore, Sebastián L. Vega, Mary M. Staehle, Peter A. Galie, Nichole M. Daringer

**Affiliations:** 1 Department of Biomedical Engineering, 3536 Rowan University, Glassboro, New Jersey 08028-1700, United States; 2 Department of Orthopaedic Surgery, Cooper Medical School of Rowan University, Camden, New Jersey United States

**Keywords:** synthetic biology, programmable synthetic receptors, cell-based devices, split fluorescent proteins, split luciferase proteins, biosensing

## Abstract

Synthetic receptors have emerged as powerful tools for precisely modulating cellular function. However, existing synthetic receptor platforms rely mainly on transcription-mediated reporting processes that are incompatible with the rapid and real-time dynamics of cellular signaling events. To address this limitation, we present SPN-FLUX (synthetic phosphorylation networks with fluorescence and luminescence expansion), a fully post-translational platform that integrates synthetic phosphorylation networks with split fluorescent or luminescent proteins, enabling rapid and tunable reporting of cellular processes. SPN-FLUX is responsive to extracellular stimuli within 1 h, providing a robust alternative to transcription-based approaches. Using mammalian cells as a model, we showcase SPN-FLUX’s versatility by designing a membrane-bound receptor that activates upon ligand-induced dimerization, as well as a constitutively active intracellular biosensor. We further validate SPN-FLUX’s biosensing capabilities by examining its responsiveness to hypoxic conditions, showcasing the ability to detect environmental changes dynamically. The modularity and programmability of SPN-FLUX establish it as a powerful platform for advancing synthetic biology and biosensing, with broad applications in both biomedical research and environmental monitoring.

## Graphical abstract

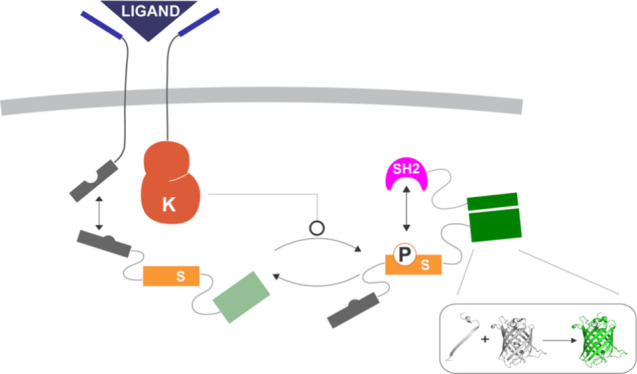

## 1 Introduction

The emergence of synthetic biology has transformed our ability to engineer and regulate biological systems with unprecedented precision. Central to this advancement are synthetic receptors, which have become essential tools across diverse applications. These engineered receptors have demonstrated significant utility in cancer immunotherapy^1^, biomarker detection^2^, and physiological monitoring^3^. In addition, the use of synthetic receptors and the idea of rewiring native inputs and outputs is expanding to applications like allergy treatment^4^, drug target identification^5^, and organic toxicity biosensing^6^. With the growing range of applications, the demand for enhanced and more adaptable synthetic receptor platforms continues to grow.

The success of these applications hinges on the design and optimization of the synthetic receptor itself. Synthetic receptors typically mimic native receptors found in nature but introduce orthogonality, modularity, and programmability by exchanging the extracellular or intracellular signaling domain based on the application. Several platforms that include chimeric antigen receptors (CARs), synthetic Notch (SynNotch) receptors, generalized extracellular molecular sensor (GEMS), and modular extracellular sensor architectures (MESA) have been widely employed as a base for synthetic receptors and re-engineered to enhance efficacy.^7–9^ While these platforms often utilize scFvs or alternatives like nanobodies for their extracellular binding domains because of their high stability and affinity, they differ in signal transduction mechanisms: CARs and SynNotch receptors typically engage cell-surface targets via a single transmembrane domain, whereas MESA and GEMS utilize dual membrane dimerization to bind soluble ligands. Recently, *Yang et al.* described a new platform that introduces orthogonality to native phosphorylation and dephosphorylation cascades, which are among the most prevalent post-translational modifications in eukaryotic cells^10^. Previously, the only work in synthetic phosphorylation cascades was in bacteria, where the WNK kinase pathway was synthetically activated^11^, but no current work has demonstrated this functionality in mammalian cells. Introducing orthogonality into one of the most prevalent post-translational modifications allows for precise control of cellular regulation, influencing key functions such as metabolism, growth, and differentiation in human cells. Yet, current manipulation of synthetic phosphorylation cascades rely on transcription-based mechanisms for signal detection and reporting, which introduces delays and reduces the ability to monitor signaling events in real time.

Fluorescent and bioluminescent proteins remain among the most widely used reporters for quantifying gene expression^12^. To enhance their utility, engineered variants have been developed wherein the reporter protein is split into two fragments that reassemble into a functional reporter—a technique known as bimolecular fluorescence complementation.^13,14^ This technique enables rapid, real-time monitoring without the lag associated with transcriptional activation and has been extensively employed for protein labeling, studying protein–protein interactions, and *in vitro* or *in vivo* imaging. Despite these advances, integration of split reporters with transmembrane receptors as post-translational sensors has been limited. Previous studies have leveraged split fluorescent proteins for tagging proteins on the nuclear envelope^15,16^ and for examining receptor oligomerization in systems such as GPCRs^17^, yet their application within synthetic transmembrane receptor frameworks remains largely unexplored.

In this study, we introduce SPN-FLUX (Synthetic Phosphorylation Networks with Fluorescence and Luminescence Expansion), a fully post-translational platform that integrates synthetic phosphorylation networks with split fluorescent or luminescent proteins. SPN-FLUX differs from existing approaches by relying solely on post-translational mechanisms for signal detection and reporting, which minimizes delays and enhances the ability to monitor signaling events in real-time. Unlike traditional synthetic receptors that depend on entire native signaling pathways for amplification, SPN-FLUX can operate either as a membrane-bound receptor that activates upon ligand-induced dimerization or as a constitutively active biosensor. Using two measurement modalities, a flow cytometer and a microplate reader, we showcase its versatility, facilitating the sensing, processing, and reporting of signaling events within 1 h and ensuring real-time detection of cellular responses in mammalian cells. Additionally, we highlight the modularity of SPN-FLUX by incorporating phosphatases, demonstrating how native components of this signal transduction cascade can be introduced to mimic real biological events and enhance the dynamic range of the system. Finally, we demonstrate the adaptability of SPN-FLUX for biosensing by modifying it to sense and respond to hypoxic conditions, thereby underscoring the modularity and programmability of this platform.

## 2 Results and Discussion

### 2.1 SPN-FLUX Design

To create a versatile, fully post-translational platform, we integrated synthetic phosphorylation networks with split fluorescent proteins. This platform is characterized by three indispensable components: a kinase active domain, a substrate, and a protein binding domain. However, it is highly programmable and can either be coupled to membrane proteins for activation by an extracellular ligand or used exclusively intracellularly. For membrane-coupled networks (Figure 1A), the FKBP/FRB (FK506 and rapamycin binding protein/FKBP–rapamycin binding protein) extracellular ligand binding platform was used to induce rapamycin-mediated dimerization^18^. FKBP and FRB were fused to the transmembrane region of CD28, commonly used in CARs, and separated with a 20 amino acid glycine-serine (GS) rich linker for flexibility^1^. Internally, one of the two receptors was linked to half of a leucine zipper (black rectangle in Figure 1A) connected with a 20 amino acid flexible linker, herein referred to as the ZC (zipper-chain) half^19^. In contrast, the other receptor, referred to as KC (kinase-chain), contains only the active protein kinase domain of the well-established kinase ABL1 (Tyrosine-protein kinase ABL1) linked to the same transmembrane region and flexible linker^20^. ABL1, a nonreceptor tyrosine kinase, is recognized for its essential role in maximizing T-cell receptor signaling and shares high homology within its kinase domain with Src-family kinases, making ABL1 ideal for initiating a synthetic phosphorylation cascade^21^. Although ABL1 contains multiple domains and exhibits polyfunctional capabilities, only amino acid residues 218–511 were used because they include the protein kinase domain and kinase activation loop, and exclude other domains such as the SH2 and SH3 domains, which are known to impart additional functions.^22,23^ The substrate component consists of three repeated immunoreceptor tyrosine-based activation motifs (ITAMs) derived from domain three of the CD3ζ protein, fused to the corresponding half of the ZC leucine zipper. Utilizing cognate leucine zipper halves on both the receptor (LZ-EE) and substrate (LZ-RR) ensures the recruitment of the substrate to the membrane due to high affinity and specificity. However, phosphorylation of substrate by membrane-bound ABL1 only takes place when they are in proximity, which occurs upon ligand-mediated dimerization. The protein binding domain (PB) is recruited to the substrate upon phosphorylation. Phosphorylated ITAMs recruit proteins with SH2 (Src Homology 2) domains; specifically, tandem phosphorylated ITAMs in CD3ζ recruit Zap70 ^24^. The protein-binding domain contains the tandem SH2 domains linked by interdomain A of Zap70. Inspired by the brightness and versatility of self-complementing split fluorescent proteins, we sought to adapt this platform to reconstitute the split fluorescent protein mNeonGreen2 ^25^. To do this, we fused the N-terminus (β-strands 1–10) of mNeonGreen2 to the substrate and the C-terminus (β-strand 11) to the protein-binding domain. We used a 10 amino acid flexible spacer (GSGSGSGSGS) to separate the split fluorescent protein from the substrate and protein-binding domain without hindering complementation. mNeonGreen2 was selected over mCherry2, and used herein, due to its higher complementation efficacy and lower background ratio (Figure S1). In addition to the membrane-coupled post-translational device, we also constructed a constitutively active and ligand-independent platform (Figure 1B). Here, we removed the ABL1 kinase from the receptor (KC) and instead fused it together with a GS-rich linker and a leucine zipper to create a subcellular and constitutively active device, hereafter referred to as KZ. The incorporation of these synthetic components did not reduce the viability of the HEK293 cells used to validate this platform. Quantification of total recorded events and single cells from flow cytometry analysis revealed no significant differences between a transfection control (miRFP270 only) and the full ligand-dependent SPN-FLUX platform (Figure S2), suggesting that circuit incorporation did not adversely affect cell growth.

**Figure 1:**
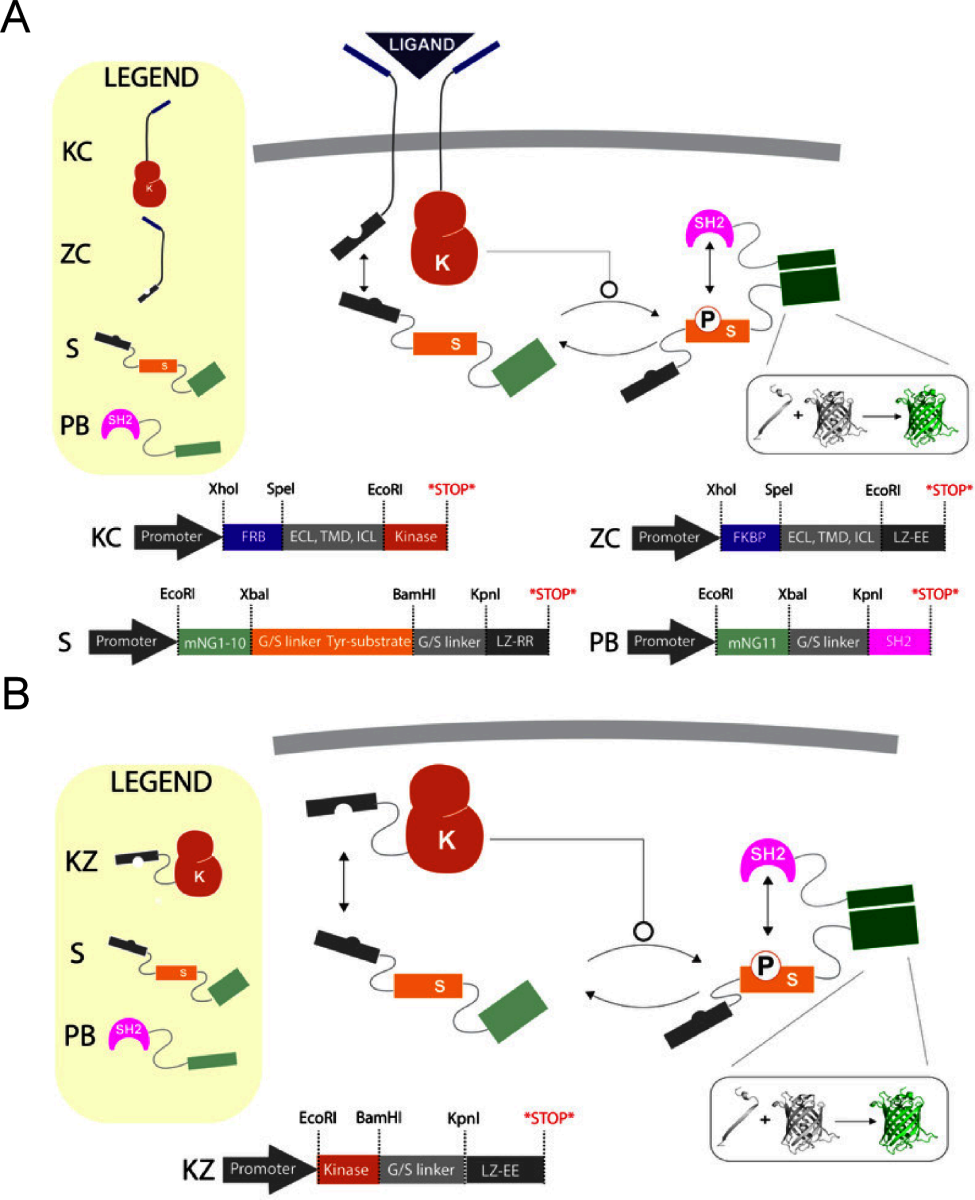
Design of SPN-FLUX and corresponding plasmid maps. A) Membrane-bound synthetic phosphorylation networks are coupled to split fluorescent proteins, activated by the binding of an extracellular ligand. The receptor-bound SPN-FLUX functions as a dimerization-based synthetic receptor targeting rapamycin (labeled “ligand”) and comprises an FKBP/FRB extracellular binding domain, a CD28-derived transmembrane domain, and either a leucine zipper (ZC) or a protein kinase (KC) domain as the intracellular signaling domain. Intracellularly, components include a substrate piece (S) and a protein-binding domain piece (PB). The substrate piece is fused with GS-rich linkers to the corresponding leucine zipper half and a split fluorescent protein, mNeonGreen2. The protein-binding domain is fused to the other half of the split fluorescent protein, mNeonGreen2. Binding of an extracellular ligand induces heterodimerization, bringing together the membrane-bound half of the leucine zipper and the kinase. This proximity, along with the split leucine zippers, allows the kinase to phosphorylate the substrate. Following phosphorylation, the protein-binding domain can bind to the phosphorylated substrate, leading to the reconstitution of the two halves of the split fluorescent protein. B) Constitutively active and ligand-independent synthetic phosphorylation networks are coupled to split fluorescent proteins. The membrane is bypassed by fusing the leucine zipper and the protein kinase directly with a GS-rich linker (KZ).

### 2.2 SFPs Maintain Signal-to-noise Ratio As Post-translational Reporters

One limitation of using self-assembling SFPs as a reporter is nonspecific complementation.^26^ Thus, our initial aim was to assess the signal-to-noise ratio of each platform. We incrementally introduced each constituent of SPN-FLUX and monitored reporter activation using both a flow cytometer and a microplate reader. We chose these instruments for their respective strengths: flow cytometry allows precise analysis at the single-cell level, reducing the influence of cell density, while the microplate reader offers a noninvasive, cost-effective approach commonly used in biosensing. Receptor-coupled networks exhibited low background on both the flow cytometer (Figure 2A) and the microplate reader (Figure 2B), with significant differences observed in samples with the full SPN-FLUX platform treated with rapamycin compared to those without. The mean fluorescence intensity (MFI) measured on the flow cytometer was normalized to an internal transfection control to mitigate variations in background noise and nonspecific effects, facilitating comparison across experiments. As no internal control could be used on the plate reader, all components of the platform were compared relative to each other. Receptor-coupled networks showed a high signal-to-noise ratio, prompting an assessment of the constitutively active intracellular device, which demonstrated low background with significant differences observed between each component compared to the full device (Figure 2C). This observation was further supported by the cell count analysis (Figure 2 **D-F**) and fluorescent microscopy (Figure 2 **G-I**) and was consistent with findings in receptor-coupled networks (Figures S3–S4). However, a limitation of using flow cytometry and normalizing to an internal control is the exclusion of cells in Q4 that are negative for the control but positive for our platform. Incorporating the cells from Q4 in Figure 2F increased MFI by 53%, but this also raised the background 13-fold due to the small number of cells (<50) in Q4 in Figure 2E. Hence, the choice of how reporter activation is monitored should be based on the specific application. Overall, this comparative analysis of individual components demonstrates that SFPs can be used as a reporter in this platform with little nonspecific complementation.

**Figure 2:**
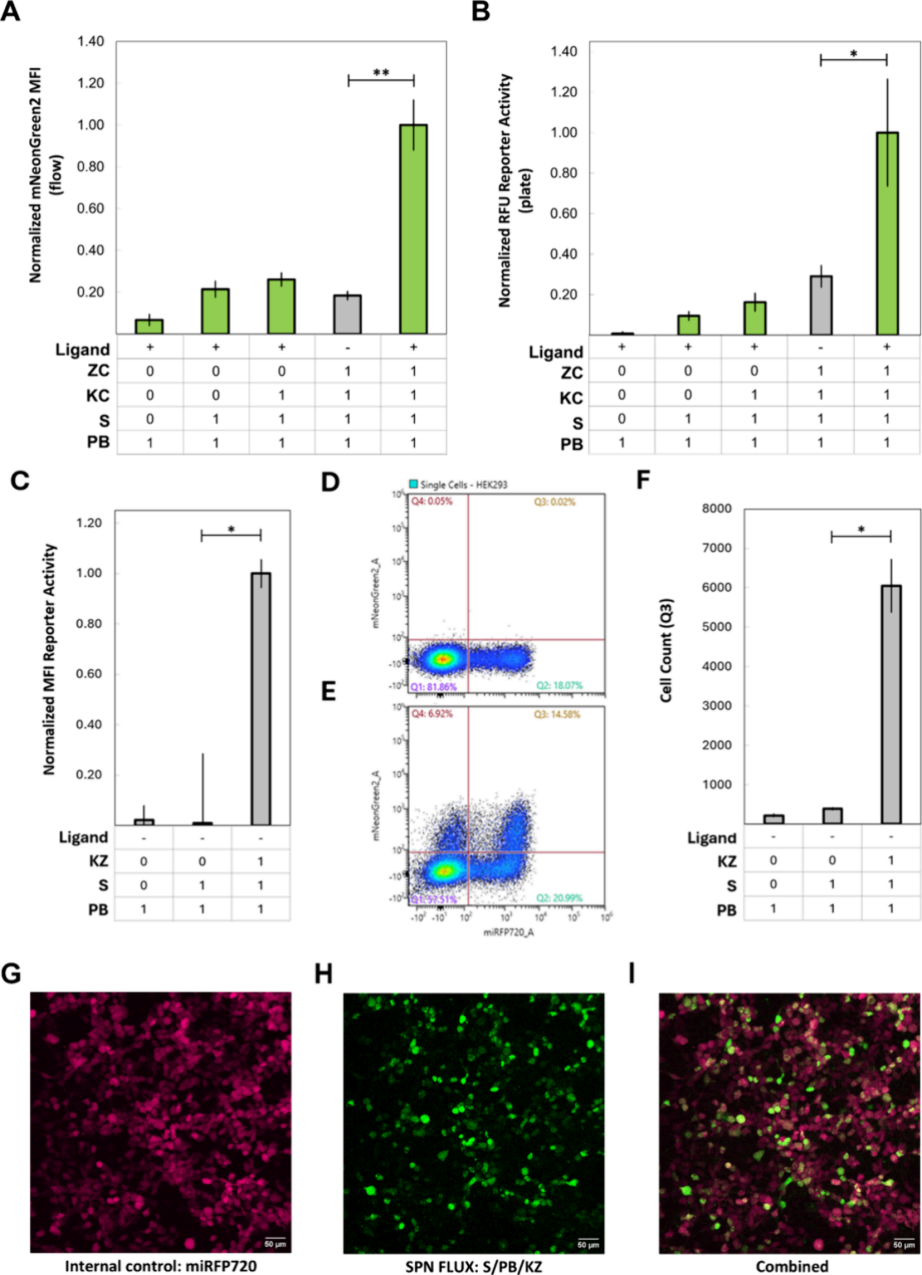
Validation of split fluorescent-coupled synthetic phosphorylation networks. A) Contribution to network signaling of each membrane-bound platform component with split fluorescence and activated by rapamycin as measured with flow cytometry and B) with a microplate reader. C) Contribution to network signaling of each component of the constitutively active ligand-independent platform measured with flow cytometry. D) Flow cytometry gating of internal transfection control (Q2) versus untransfected cells (Q1). E) Flow cytometry gating of untransfected cells (Q1), cells transfected with just the internal control (Q2), cells transfected with the internal control and constitutively active ligand-independent platform (Q3), and cells transfected without the internal control but with the constitutively active ligand-independent platform (Q4). F) Comparison of cell count using cells transfected with the internal control and the constitutively active ligand-independent platform (Q3). G) Fluorescent microscopy of internal control (miRFP720 fluorescence, corresponding to Q2 and Q3), H) constitutively active ligand-independent platform (mNeonGreen2, corresponding to Q3 and Q4), and I) combined z-stack. Gray bars represent conditions without ligand addition, while green bars indicate ligand addition for experiments using split fluorescent proteins. Error bars represent standard deviation and each experiment was performed in biological triplicate (*p ≤ 0.05, **p ≤ 0.01). ZC = zipper chain, KC = kinase chain, K = kinase, S = substrate, PB = protein binding, KZ = kinase zipper.

### 2.3 SPN-FLUX Maintains Native Dynamics

Phosphorylation serves as a key regulator of signal transduction due to its rapid and reversible nature^27^. Yang et al. demonstrated that phosphorylation of the substrate can be detected in as little as 15 min^10^. Thus, we compared the kinetics of the SPN-FLUX platform, labeled in Figure 3A as “membrane-dependent” with a ligand-inducible cytosolic device, created by directly fusing FRB to the N-terminus of mNeonGreen2–11 and FKBP to the C-terminus of mNeonGreen2–1–10, referred to as “membrane-independent” in Figure 3B. Hemagglutinin (HA) tagging of the FRB receptor provided means to verify that the ZC component was on the cell surface in the membrane-dependent scenario. Figure S5 indicates a significant difference in both the percentage of positive cells and the mean fluorescence intensity compared to the transfection control in cells expressing the ZC construct. Both the membrane-dependent and membrane-independent systems were dosed with rapamycin, which is cell permeable and capable of reaching both cell surface and intracellular receptors, and fluorescence was measured at regular intervals over a period of 60 min. Figure 3C reveals similar kinetics between both conditions, with a substantial increase in fluorescence within an hour of dosing. The membrane-independent featured significantly higher levels of activation for the first 10 min compared to the membrane-dependent condition, indicating that the dynamics of KC-mediated phosphorylation and PB binding to the phosphorylated substrate caused a short delay in fluorescence compared to the intracellular receptor dimerization. Similar trends were observed using the microplate reader, with significant fluorescence differences observed at 1 h (Figure S6). Comparison with typical harvest times (24 h post ligand addition) highlighted the pronounced response, with a significant difference between 1 and 24 h, likely attributable to the stability and accumulation of reporter activity under a constitutive promoter (Figure S7). Future work is needed to investigate the long-term dynamics of the system once the ligand is removed or degraded to determine the reversibility of the synthetic network. Nonetheless, these results underscore that the addition of SFPs allows for a rapid and dynamic response, verifying that fluorescence did not exhibit slower kinetics compared to phosphorylation due to reassembly mechanics (such as chromophore maturation).

**Figure 3:**
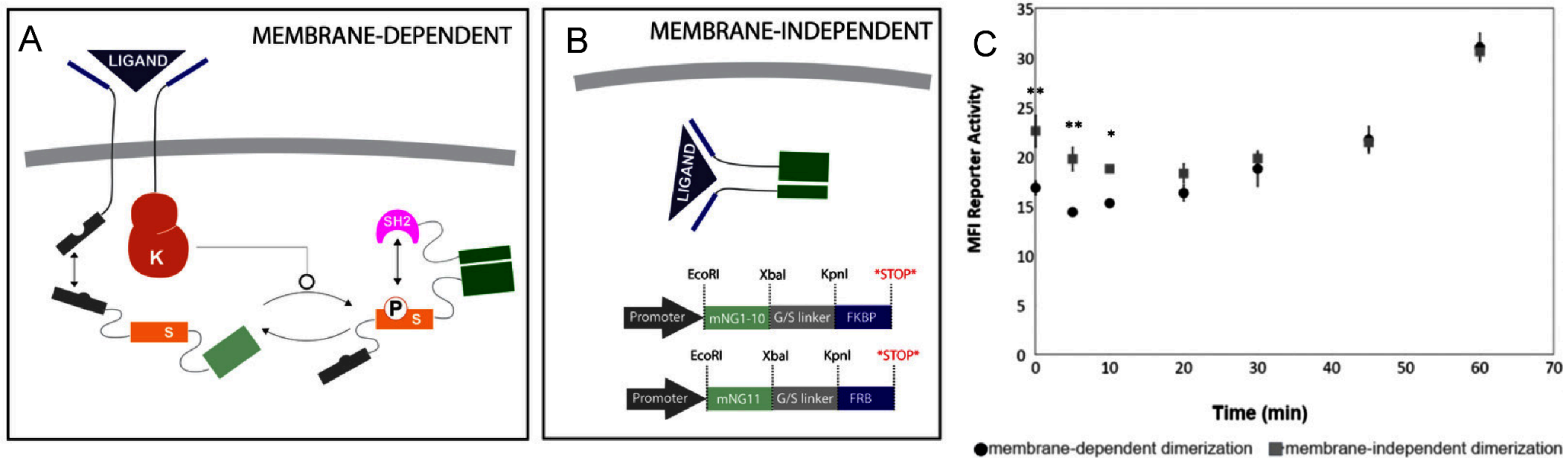
Kinetics of SPN-FLUX in membrane-dependent (A) and cytosolic, membrane-independent (B) configurations. C) Time course illustrating the membrane-dependent platform with split fluorescence within the first 60 min post rapamycin addition. The membrane-independent approach fused the FRB/FKBP ectodomain directly to mNeongreen2 β-strands 1–10/11, respectively, to simulate direct dimerization. Mean fluorescent intensity was measured with flow cytometry. Plasmid maps for the membrane-independent constructs are provided in panel (B). (*p ≤ 0.05, **p ≤ 0.01).

### 2.4 Modularity and Adaptability of SPN-FLUX

A fundamental challenge in synthetic receptor design is the lack of dynamic control, a limitation that synthetic phosphorylation networks (SPNs) seek to address^28^. Given that phosphorylation is a reversible process, while the reconstitution of SFPs is irreversible, we aimed to explore whether native phosphatases could provide a means of tuning the system by increasing the dynamic range. Among the protein tyrosine phosphatases (PTPs) known to negatively regulate the T-cell response, PTP1B emerges as a central nonreceptor PTP^29^. Therefore, we fused the N-terminus catalytic domain of PTP1B with the same leucine zipper used in ZC and KZ, designated herein as PZ (phosphatase-leucine zipper). The introduction of PZ was designed to competitively bind to the substrate, thereby diminishing its phosphorylation by reducing its interaction with KC in addition to facilitating substrate dephosphorylation upon contact (Figure 4A). The incorporation of PZ into the device led to a substantial reduction in fluorescence, bringing it to levels approaching background and even falling below levels observed when the ligand was removed (Figure 4B). These findings underscore the effectiveness of integrating SFPs as reporters into the device, while also demonstrating that phosphatases can be introduced as a viable control mechanism to attenuate the system response.

**Figure 4:**
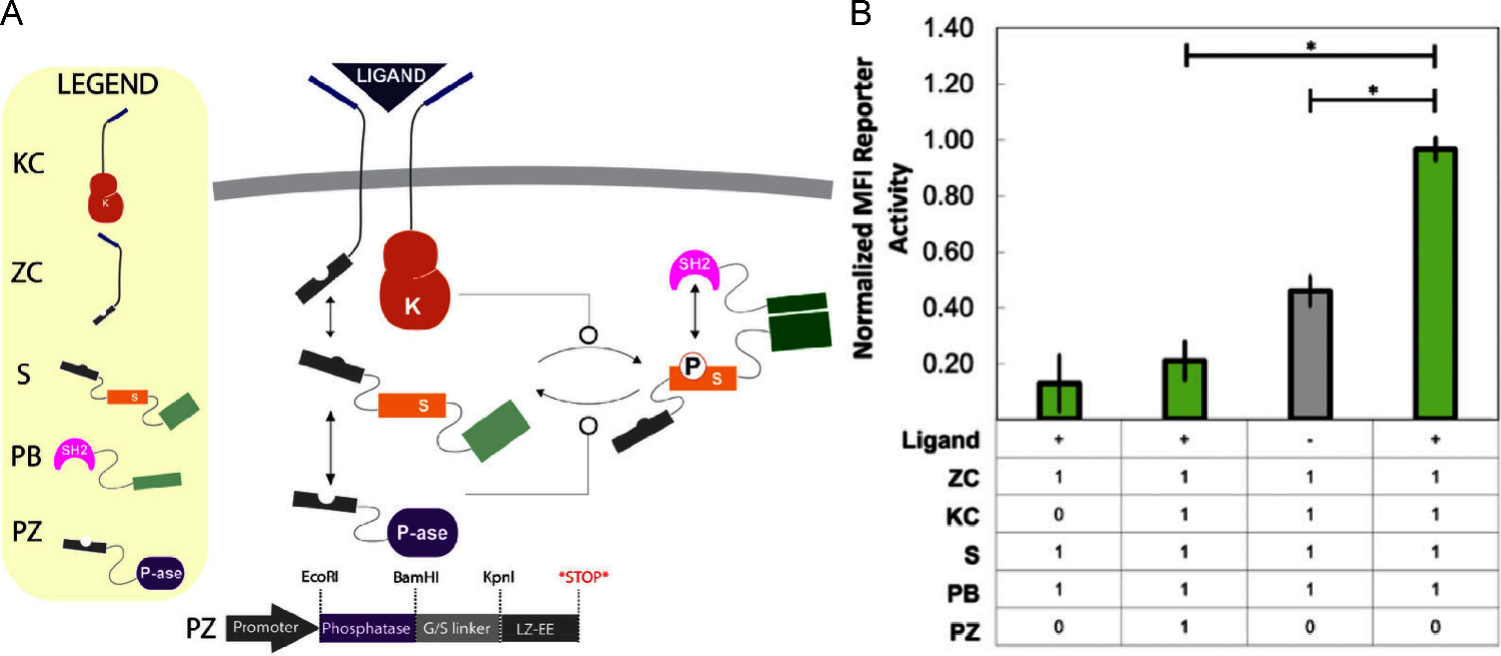
Modularity and programmability of SPN-FLUX demonstrated with phosphatase-driven regulation. A) Schematic of the platform introducing system control by fusing a phosphatase to a leucine zipper (PZ). B) Contribution of the phosphatase introduced to the membrane-bound platform with split fluorescence, showing a reduction in fluorescence in the presence of the ligand as measured by flow cytometry. (*p ≤ 0.05)

Building upon this modular framework, we explored the potential of SPN-FLUX for more practical biosensing applications. We hypothesized that a constitutively active, ligand-independent variant of SPN-FLUX could serve as a biosensor. To test this hypothesis, we developed a hypoxia-responsive version of SPN-FLUX, leveraging the oxygen-dependent degradation domain (ODD) of hypoxia-inducible factor 1-alpha (HIF-1α). Hypoxia sensing plays a critical role in cell-based therapies and molecular imaging for tumor detection.^30–33^ In normoxic conditions, proline hydroxylation within the ODD domain targets HIF-1α for degradation via the ubiquitin-proteasome pathway^34^. Therefore, the HIF-1α ODD presents an ideal regulatory element for integration with our constitutively active, ligand-independent SPN-FLUX, enabling hypoxia-responsive biosensing. To develop a hypoxia-sensing variant of SPN-FLUX, we fused a truncated version of the HIF-1α ODD, as described by Juillerat et al., to the substrate, enabling SPN-FLUX activation and fluorescence under hypoxic conditions while ensuring its degradation in normoxia^32^ (Figure 5A). After 24 h in 1% oxygen, we observed a significant, 5-fold increase in mean fluorescence intensity (MFI) compared to normoxia (Figure 5B). Furthermore, the hypoxia-responsive SPN-FLUX exhibited a significant difference in fluorescence compared to the nonhypoxia-responsive control (substrate without the ODD domain), further highlighting its potential for hypoxia sensing applications. Representative scatter plots are provided in Figure S9. Incorporation of the ODD to endow SPN-FLUX with oxygen-sensing capabilities demonstrates that the system is responsive to a more physiologically relevant stimulus than rapamycin, given the importance of hypoxia in pathological processes including those within the tumor microenvironment. Future studies will evaluate this response at shorter time scales to interrogate whether the degradation of the ODD element yields similar dynamics as the receptor-mediated phosphorylation modality used to respond to rapamycin.

**Figure 5:**
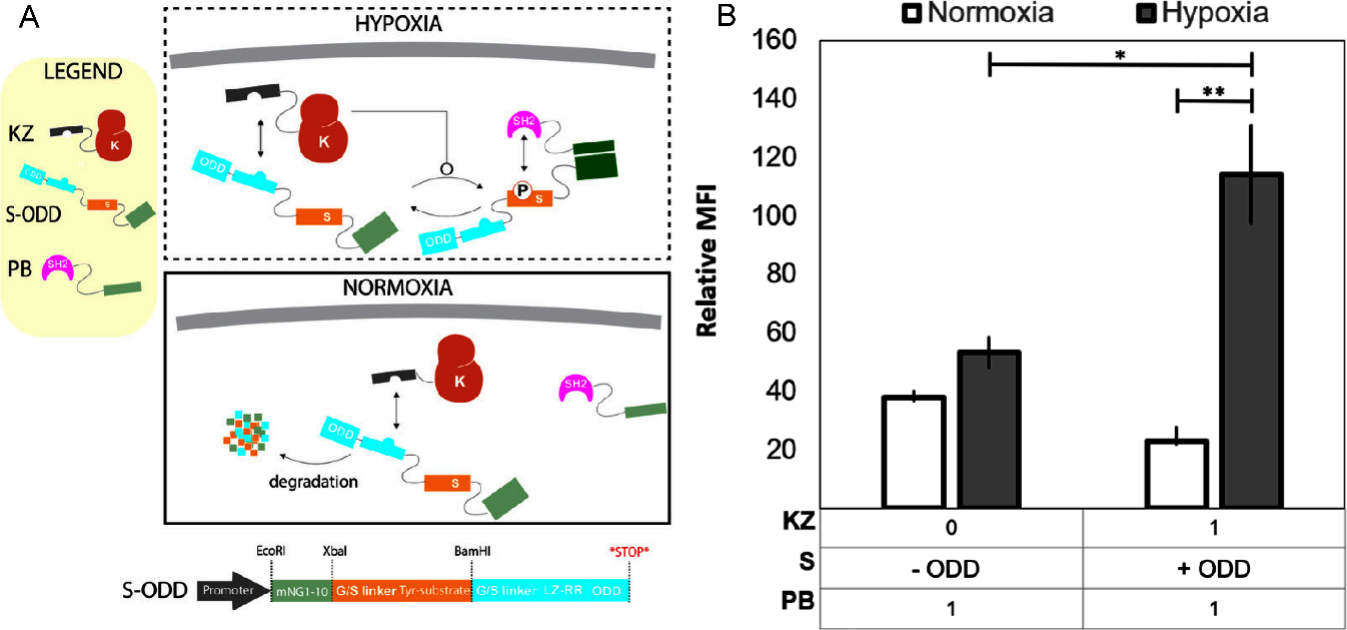
SPN-FLUX as a hypoxia biosensing system. A) Schematic of SPN-FLUX adaptation for hypoxia sensing. The constitutively active platform incorporates the HIF-1α ODD domain fused to the substrate, enabling degradation under normoxic conditions and stabilization in hypoxia. B) Hypoxia-sensing SPN-FLUX was subjected to 24 h in either 1% (hypoxia) or atmospheric oxygen (normoxia) levels, and MFI was measured using a flow cytometer in cells transfected with the substrate construct with and without ODD domain fusion. (*p ≤ 0.05, **p ≤ 0.01).

Beyond tunable control, modularity is a critical feature of synthetic receptors. To demonstrate the adaptability of SPN-FLUX for nonfluorescence-based applications, we replaced SFPs with split luciferase proteins (SLPs), specifically firefly luciferase, which is widely used in mammalian systems. Analogous to the SFP-based platform, we fused the N-terminus of firefly luciferase to the substrate and the C-terminus to the protein-binding domain, separated by the same 10 amino acid flexible linker^35^. We observed minimal background when evaluating the signal-to-noise ratio of the membrane-bound system employing SLPs (Figure 6A). Given that SLPs share reassembly mechanisms with SFPs but incorporate an exogenous substrate, D-Luciferin, we conducted a time course to determine the kinetics of split firefly luciferase-mediated luminescence (Figure 6B). Comparable to SFP usage, significant increases in luminescence were detected within an hour, and the 24 h time point was significantly higher than the 6 h measurement. Overall, these results suggest that SLPs can serve as a post-translational reporter for SPN-FLUX. Subsequently, we introduced PZ into our SLP-coupled device to evaluate its potential to regulate dynamics in this system (Figure 6C). We observed a reduction in relative luminescent units (RLU) upon adding PZ to the receptor-coupled device, but in contrast to the SFP system, the levels remained significantly higher than the nonligand conditions. We suspect the apparent reduction in phosphatase effectiveness may partly result from the microplate reader, as it does not exclude cells lacking PZ; in contrast to SFP, where target fluorescence is measured within a gated, transfected cell population. Strategies such as LucFlow and stably expressing cell-lines may address this issue^36^. Nonetheless, these findings highlight the potential of SLPs as post-translational reporters within SPN-FLUX, further underscoring the platform’s high modularity and broad applicability.

**Figure 6:**
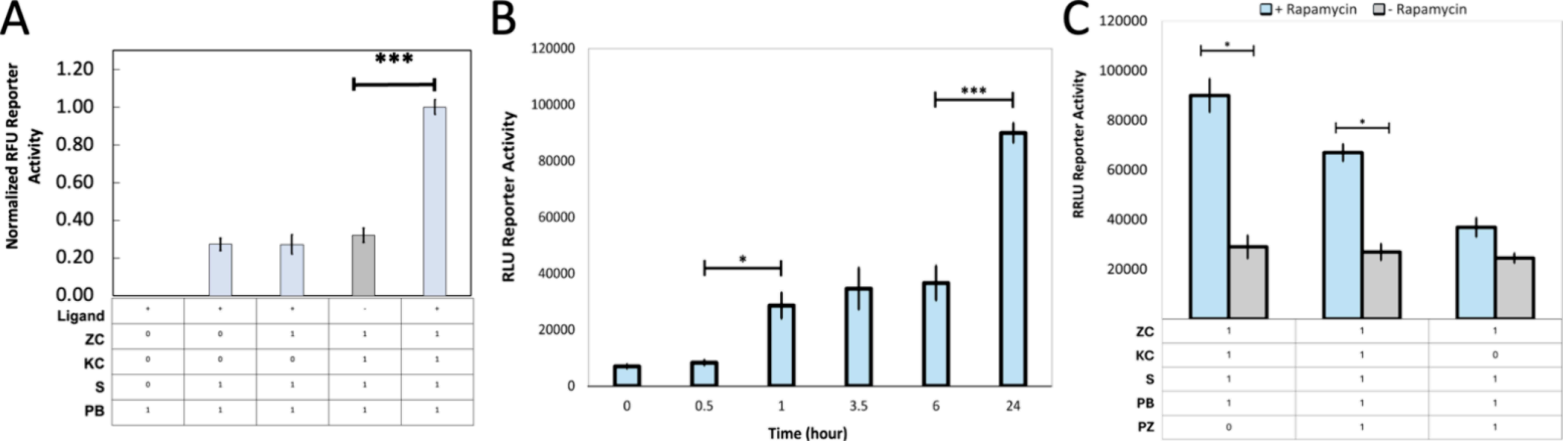
A) Contribution to network signaling of each membrane-bound platform component with split firefly luciferase, activated by rapamycin and normalized to an empty vector plasmid. B) Time course illustrating the membrane-dependent platform with split firefly luciferase over 24 h postrapamycin addition. C) Comparison of PZ introduced to the membrane-bound platform with split luciferase on network signaling. Gray bars represent conditions without ligand addition, while blue bars indicate ligand addition for experiments using SLPs. Error bars represent standard deviation, and each experiment was performed in biological triplicate. Significance was calculated using posthoc Tukey tests between different conditions (*p ≤ 0.05, **p ≤ 0.01, ***p ≤ 0.001). ZC = zipper chain, KC = kinase chain, K = kinase, S = substrate, PB = protein binding, KZ = kinase zipper.

## 3 Conclusions

In this study, we present SPN-FLUX, a novel platform that leverages SFPs or SLPs as post-translational reporters to enhance the functionality of recently described synthetic phosphorylation networks^10^. By integrating these split reporters into a synthetic phosphorylation cascade, we expand the applicability of these systems, enabling their use in diverse biomedical and environmental applications. Specifically, we have engineered split fluorescent and luminescent proteins to associate with the substrate and protein-binding domain of the phosphorylation cascade, facilitating activation upon extracellular ligand binding or constitutive activation without external input. This design preserves the rapid, real-time detection of phosphorylation events, providing a robust alternative to transcription-based platforms by circumventing the inherent delays associated with gene expression. Unlike transcriptional systems, which require transcription, translation, and protein accumulation for signal generation, SPN-FLUX produces detectable outputs within an hour, enabling faster responses and dynamic regulation while preserving the inherent speed of phosphorylation cascades. This functionality is crucial for detecting transient environmental stimuli like hypoxia, as demonstrated by the results.

The flexibility and modularity of the SPN-FLUX platform are key advantages that make it a powerful tool for a wide array of applications. Its compatibility with both flow cytometry and microplate readers, combined with the interchangeable use of split fluorescent or luminescent reporters, enhances its versatility as an analytical tool and allows for customization based on specific experimental needs. We demonstrate this adaptability by substituting split fluorescent proteins with SLPs, providing an alternative detection modality. The incorporation of the PZ and ODD fragments provide control over signaling outputs, a critical feature for applications requiring dynamic regulation. Future perspectives of this platform lie not only within those adaptations, but also in the creativity of users. A part-based system like SPN-FLUX offers modularity to build-in and employ logic gate scenarios and potentially even integration with native signaling to control cell function. These future applications will also require validation of the SPN-FLUX within more physiologically relevant cell types than the HEK293-T cells used in this study. Nonetheless, by leveraging split fluorescent and luminescent proteins as post-translational reporters, SPN-FLUX offers a powerful alternative to transcription-based signal amplification, enabling rapid, real-time detection while preserving the inherent speed of signal transduction. This platform integrates the efficiency of post-translational control with the precision and adaptability required of modern synthetic biology applications.

## 4 Methods

### 4.1 Plasmid Generation

The Supporting Information includes the amino acid sequences for all constructs used in this study (Table S1 and S2), along with their corresponding plasmid maps. Plasmid construction, as described in Yang et al. was achieved by standard molecular cloning procedures.^10^ In addition, firefly luciferase was split via polymerase chain reaction (PCR) amplification, all primers used are included in Table S3.35 All DNA constructs were synthesized and inserted into the vector plasmid pcDNA GNSTM-3-RVG-10-Lamp2b-HA (Addgene plasmid 71294) using restriction digestion, restriction enzymes are included in the corresponding plasmid maps.^37^ All subfragments were sequenced to ensure accurate construction.

### 4.2 Cell culture and Transfection

HEK293-T cells (ATCC CRL-1573) were maintained in 21% oxygen and 5% CO_2_ and cultured with Dulbecco Modified Eagle Medium (DMEM) with 10% fetal bovine serum (FBS), 4 mM l-glutamine and 1% penicillin-streptomycin. Transient transfection using the CaCl_2_–HEPES-buffered saline (HeBS) method was used for all experiments except for validation of surface expression, for which Lipofectamine was used. Cells were plated in 24-well plates at a density of 7.5 × 10^4^ cells per well and transfected 24 h later. 24 h post-transfection, the transfection medium was removed and replaced with fresh medium. For all experiments, the total DNA per cell was kept at 550 ng, with each component of the platform assigned a specific amount. The internal transfection control (miRFP720) was used at 175 ng, each receptor half (ZC and KC) at 100 ng, the substrate at 100 ng, the protein-binding domain at 75 ng, and the phosphate domain at 75 ng. For experiments involving cell surface coupling, the fresh media was supplemented with either 100 nM rapamycin (MP Biomedicals 02159346-CF) or an equivalent volume of DMSO (see Figure S8A for concentration curve). Cells were treated with rapamycin 24 h post-transfection and cultured for another 24 h (48 h post-transfection) before harvesting and analysis via flow cytometry or microplate reader. To maintain consistent cell density throughout the time course experiments, rapamycin was administered during media change, and cells were harvested at 48 h post-transfection for the 24 h time point analysis. For other time points, rapamycin was added 48 h post-transfection followed by the necessary incubation period before harvest. For hypoxia-sensing SPN-FLUX experiments, cells were plated in 24-well plates at a density of 7.5 × 10^4^ cells per well and transfected 24 h later. 24 h post-transfection, cells were subjected to either atmospheric or 1% oxygen (Coy O2 Control InVitro Cabinet) for 24 h before harvesting and analysis via flow cytometry.

### 4.3 Flow Cytometry

Transfected cells were harvested using phosphate-buffered saline (PBS) supplemented with 0.5 mM EDTA (Ethylenediaminetetraacetic acid) 24 h postmedium change and subsequently suspended in PBS containing 2% BSA (bovine serum albumin). The cell suspensions were immediately analyzed using a flow cytometer (SONY SA3800) or a microplate reader (refer to the equipment specifications below). Similarly, for hypoxia-sensing SPN-FLUX experiments, cells were removed from the hypoxic chamber 24 h after transfection and immediately harvested and analyzed using the same methods previously mentioned. Data analysis was conducted utilizing Sony Biotechnologies’ proprietary analysis software, SA3800 Spectral Analyzer. Single cells were isolated from cell aggregates and debris based on forward scatter (FSC) and side scatter (SSC) gating parameters. Transfected cells were established through gating on the internal fluorescent transfection control, miRFP720, similar to MESA^7^. And mNeonGreen2 mean fluorescent intensity (MFI) was considered within the transfected miRFP720+ population.

### 4.4 Microplate Reader

Fluorescence and luminescence emissions were quantified using a 96-well Spectramax ID5 plate reader. Excitation of the fluorescent protein, mNeonGreen2, was excited at 506 nm and emissions were measured at 517 nm. The luminescence emitted by Firefly luciferase was measured across all wavelengths. Cells were harvested with the same protocol as flow cytometry experiments. D-luciferin (Invitrogen L2916) was introduced at a concentration of 1 mg/mL (refer to Figure S8B for the concentration curve) to the suspended cells in the 96-well microplate 15 min prior to reading luminescence.

### 4.5 Fluorescent Microscopy

Cell imaging was conducted 48 h post-transfection using a Nikon Eclipse Ti Confocal Microscope to visualize network activation. The far-red (640 nm) and green (488 nm) channels were employed to observe the internal transfection control and reconstitution of mNeonGreen2, respectively. A max intensity z-stack was curated before color channels were merged for analysis of network dynamics.

### 4.6 Statistical Analysis

All experiments were performed in biological triplicate and repeated to ensure robust statistical analysis. For flow cytometry experiments, mNeonGreen2MFI was measured within the transfected population for each sample and normalized to the mNeonGreen2MFI measurement within cells only containing the internal transfection control, miRFP720. Furthermore, a minimum threshold of 10,000 cells per sample was established to ensure reliable statistical analysis. In microplate reader experiments, relative fluorescent units (RFU) and relative luminescent units (RLU) were measured for each sample and normalized to an empty plasmid vector, if applicable. Error bars throughout represent standard deviation across triplicate samples. RStudio was used to run one and two-way ANOVAs depending on the experiment, and posthoc Tukey tests were used to evaluate significant differences between conditions. Two sample *t* tests were used to determine significance between individual treatments (e.g., ± ligand in receptor-mediated SPN-FLUX and ± KC in constitutively active version in Figure 2 and ± hypoxia in Figure 5) Standard deviation of the fold change was calculated using error propagation. An example, using the raw data, is provided below to illustrate how the standard deviation of the fold change (1.40) was computed for the substrate and protein binding fold change mean fluorescence intensity (MFI) between hypoxia and normoxia conditions. Significance levels are denoted as follows when shown: *p ≤ 0.05, **p ≤ 0.01, ***p ≤ 0.001.

**Variables**

Hypoxia MFI: M_Hypoxia_ = 192.52, SD = σ_Hypoxia_ = 5.08

Normoxia MFI: M_Normoxia_ = 177.25, SD = σ_Normoxia_ = 2.20

Background (Internal transfection control of miRFP720) MFI: M_bg_ = 137.63, SD = σ_bg_ = 4.51

**Background Correction**

Background-corrected Hypoxia MFI: M_Hypoxia′_=M_Hypoxia_–M_bg_ = 192.52–137.63 = 53.15

Background-corrected Normoxia MFI: M_Normoxia′_=M_Normoxia_–M_bg_ = 177.25–137.63 = 37.87

Fold Change (FC): FC = M_Hypoxia_/M_Normoxia′_ = 53.1537.87 = 1.4

**Propagation of Errors**

$$\sigma_{Hypoxia}^{\prime }=\sqrt{\sigma_{Hypoxia}^{2}+\sigma_{bg}^{2}}=\sqrt{{\left(5.08\right)}_{Hypoxia}^{2}+{\left(4.51\right)}_{bg}^{2}}=6.80$$

$$\sigma_{Normoxia}^{\prime }=\sqrt{\sigma_{Normoxia}^{2}+\sigma_{bg}^{2}}=\sqrt{{\left(2.20\right)}_{Hypoxia}^{2}+{\left(4.51\right)}_{bg}^{2}}=5.02$$

$$\frac{\sigma_{FC}}{FC}=\sqrt{{\frac{\sigma_{Hypoxia}^{\prime }}{M_{Hypoxia}^{\prime }}}^{2}+{\frac{\sigma_{Normoxia}^{\prime }}{M_{Normoxia}^{\prime }}}^{2}}$$

$$\frac{\sigma_{FC}}{1.4}=\sqrt{{\frac{6.80}{53.15}}^{2}+{\frac{5.02}{37.87}}^{2}};\sigma_{FC}=0.26$$

## Supplementary Material





## Notes

(Figure S1) Bimolecular fluorescent complementation (BiFC) comparison of mNeonGreen and mCherry; (Figure S2) recorded events and daughter cell population evaluated by flow cytometry; (Figure S3) SPN-Flux transfection groups’ single cell population mNeonGreen vs miRFP720 dot plot supports MFI trends in Figure 3; (Figure S4) SPN-Flux in expressed in HEK293 evaluated via fluorescence microscopy; (Figure S5) synthetic transmembrane receptors trafficked to the cell membrane, as confirmed by AlexaFluor-488-conjugated HA-antibody treatment of live cells; (Figure S6) SPN-Flux activation dynamics observed over a period of 90 min; (Figure S7) SPN-Flux activation dynamics observed over a period of 24 h for receptor-mediated and constitutively active configurations; (Figure S8) rapamycin (A) and d-luciferin concentration curves evaluated by flow cytometer and plate reader, respectively; (Figure S9) representative flow cytometry scatter plots for Figure 5 (PDF)
(Tables S1 and S2) DNA construct data used in this study (XLSX)

## Abbreviations

SPN-FLUX: (synthetic phosphorylation networks with fluorescence and luminescence expansion)
CAR: (chimeric antigen receptor)
SynNotch: (synthetic notch)
MESA: (modular extracellular sensor architecture)
GEMS: (generalized extracellular molecular sensor)
(SFPs): Split fluorescent proteins
FKBP/FRB: (FK506 and rapamycin binding protein/FKBP–rapamycin binding protein)
ZC: (zipper chain)
KC: (kinase chain)
ABL1: (Tyrosine-protein kinase ABL1)
S: (substrate)
ITAMs: (immunoreceptor tyrosine-based activation motifs)
protein-binding: (protein binding)
SH2: (Src Homology 2)
K: (kinase)
KZ: (kinase-leucine zipper)
MFI: (mean fluorescent intensity)
PTPs: (protein tyrosine phosphatases)
PZ: (phosphatase-leucine zipper)
(ODD): Oxygen-Dependent Degradation Domain
(HIF-1α): Hypoxia-Inducible Factor 1-alpha
SLPs: (split luciferase proteins)
RLU: (relative luminescent units)
PCR: (polymerase chain reaction)
DMEM: (Dulbecco’s modified eagle medium)
FBS: (fetal bovine serum)
HeBS: (HEPES-buffered saline)
PBS: (phosphate-buffered saline)
BSA: (bovine serum albumin)
FSC: (forward scatter)
SSC: (side scatter).

## References

[1] Zhang C., Liu J., Zhong J. F., Zhang X. (2017). Engineering CAR-T cells. Biomarker. Research..

[2] Chang H. J., Zúñiga A., Conejero I., Voyvodic P. L., Gracy J., Fajardo-Ruiz E., Cohen-Gonsaud M., Cambray G., Pageaux G. P., Meszaros M., Meunier L., Bonnet J. (2021). Programmable
receptors enable bacterial biosensors to detect pathological biomarkers
in clinical samples. Nat. Commun..

[3] Dolberg T. B., Gunnels T. F., Ling T., Sarnese K. A., Crispino J. D., Leonard J. N. (2024). Building Synthetic Biosensors Using
Red Blood Cell
Proteins. ACS Synth Biol..

[4] Ausländer D., Eggerschwiler B., Kemmer C., Geering B., Ausländer S., Fussenegger M. (2014). A designer cell-based histamine-specific human allergy
profiler. Nat. Commun..

[5] Kroeze W. K., Sassano M. F., Huang X. P., Lansu K., McCorvy J. D., Giguère P. M. (2015). PRESTO-Tango as an open-source resource
for interrogation of the druggable human GPCRome. Nat. Struct Mol. Biol..

[6] Chen X., Yao H., Song D., Sun G., Xu M. (2022). Extracellular chemoreceptor
of deca-brominated diphenyl ether and its engineering in the hydrophobic
chassis cell for organics biosensing. Chemical
Engineering Journal..

[7] Daringer N. M., Dudek R. M., Schwarz K. A., Leonard J. N. (2014). Modular
Extracellular
Sensor Architecture for Engineering Mammalian Cell-based Devices. ACS Synth Biol..

[8] Scheller L., Strittmatter T., Fuchs D., Bojar D., Fussenegger M. (2018). Generalized
extracellular molecule sensor platform for programming cellular behavior. Nat. Chem. Biol..

[9] Morsut L., Roybal K. T., Xiong X., Gordley R. M., Coyle S. M., Thomson M. (2016). Engineering
Customized Cell Sensing and Response Behaviors
Using Synthetic Notch Receptors. Cell..

[10] Yang X., Rocks J. W., Jiang K., Walters A. J., Rai K., Liu J. (2025). Engineering synthetic phosphorylation signaling networks
in human cells. Science..

[11] Schiapparelli P., Pirman N. L., Mohler K., Miranda-Herrera P. A., Zarco N., Kilic O. (2021). Phosphorylated
WNK kinase
networks in recoded bacteria recapitulate physiological function. Cell Rep..

[12] Soboleski M. R., Oaks J., Halford W. P. (2005). Green fluorescent
protein is a quantitative
reporter of gene expression in individual eukaryotic cells. FASEB J..

[13] Kerppola T. K. (2006). Design
and implementation of bimolecular fluorescence complementation (BiFC)
assays for the visualization of protein interactions in living cells. Nat. Protoc..

[14] Luker K. E., Smith M. C. P., Luker G. D., Gammon S. T., Piwnica-Worms H., Piwnica-Worms D. (2004). Kinetics of regulated protein-protein interactions
revealed with firefly luciferase complementation imaging in cells
and living animals. Proc. Natl. Acad. Sci. U.
S. A..

[15] Leonetti M. D., Sekine S., Kamiyama D., Weissman J. S., Huang B. (2016). A scalable
strategy for high-throughput GFP tagging of endogenous human proteins. Proceedings of the National Academy of Sciences..

[16] Shelton, S. N. , Smith, S. E. , Jaspersen, S. L. Split-GFP Complementation to Study the Nuclear Membrane Proteome Using Microscopy. In: Goldberg, M. W. editor. The Nuclear Pore Complex: Methods and Protocols [Internet]. Springer US, New York, NY; 2022 [cited 2025 Feb 19]. p 205–13. 10.1007/978-1-0716-2337-4_13. 35412240

[17] Wouters E., Vasudevan L., Crans R. A. J., Saini D. K., Stove C. P. (2019). Luminescence-
and Fluorescence-Based Complementation Assays to Screen for GPCR Oligomerization:
Current State of the Art. International Journal
of Molecular Sciences..

[18] Banaszynski L. A., Liu C. W., Wandless T. J. (2006). Characterization of the FKBP·Rapamycin·FRB
Ternary Complex [J. Am. Chem. Soc. 2005, 127, 4715–4721]. J. Am. Chem. Soc..

[19] Acharya A., Ruvinov S. B., Gal J., Moll J. R., Vinson C. (2020). ACS Publications.
American Chemical Society; 2002 [cited 2024 Mar 11]. A Heterodimerizing
Leucine Zipper Coiled Coil System for Examining the Specificity of
a Position Interactions: Amino Acids I, V, L, N, A, and K. Biochemistry.

[20] Mócsai A., Ruland J., Tybulewicz V. L. J. (2010). The SYK tyrosine kinase: a crucial
player in diverse biological functions. Nat.
Rev. Immunol..

[21] Gu J. J., Ryu J. R., Pendergast A. M. (2009). Abl Tyrosine Kinases in T-cell signaling. Immunol Rev..

[22] Lamontanara A. J., Georgeon S., Tria G., Svergun D. I., Hantschel O. (2014). The SH2 domain
of Abl kinases regulates kinase autophosphorylation by controlling
activation loop accessibility. Nat. Commun..

[23] Merő B., Radnai L., Gógl G., Tőke O., Leveles I., Koprivanacz K. (2019). Structural insights
into the tyrosine phosphorylation–mediated inhibition of SH3
domain–ligand interactions. J. Biol.
Chem..

[24] Neumeister E. N., Zhu Y., Richard S., Terhorst C., Chan A. C., Shaw A. S. (1995). Binding
of ZAP-70 to phosphorylated T-cell receptor zeta and eta enhances
its autophosphorylation and generates specific binding sites for SH2
domain-containing proteins. Mol. Cell. Biol..

[25] Feng S., Sekine S., Pessino V., Li H., Leonetti M. D., Huang B. (2017). Improved split fluorescent proteins for endogenous protein labeling. Nat. Commun..

[26] Romei M. G., Boxer S. G. (2019). Split Green Fluorescent
Proteins: Scope, Limitations,
and Outlook. Annual Review of Biophysics..

[27] Ardito F., Giuliani M., Perrone D., Troiano G., Muzio L. L. (2017). The crucial
role of protein phosphorylation in cell signaling and its use as targeted
therapy (Review). Int. J. Mol. Med..

[28] Zhao N., Song Y., Xie X., Zhu Z., Duan C., Nong C., Wang H., Bao R. (2023). Synthetic
biology-inspired cell engineering in diagnosis, treatment, and drug
development. Sig. Transduct. Target Ther..

[29] Liu R., Mathieu C., Berthelet J., Zhang W., Dupret J. M., Rodrigues Lima F. (2022). Human Protein
Tyrosine Phosphatase 1B (PTP1B): From
Structure to Clinical Inhibitor Perspectives. Int. J. Mol. Sci..

[30] Zhu X., Chen J., Li W., Xu Y., Shan J., Hong J. (2024). Hypoxia-Responsive CAR-T
Cells Exhibit Reduced Exhaustion
and Enhanced Efficacy in Solid Tumors. Cancer
Res..

[31] Iglesias P., Penas C., Barral-Cagiao L., Pazos E., Costoya J. A. (2019). A Bio-inspired
Hypoxia Sensor using HIF1a-Oxygen-Dependent Degradation Domain. Sci. Rep..

[32] Juillerat A., Marechal A., Filhol J. M., Valogne Y., Valton J., Duclert A., Duchateau P., Poirot L. (2017). An oxygen
sensitive self-decision making engineered CAR T-cell. Sci. Rep..

[33] Misra T., Baccino-Calace M., Meyenhofer F., Rodriguez-Crespo D., Akarsu H., Armenta-Calderón R., Gorr T. A., Frei C., Cantera R., Egger B., Luschnig S. (2016). A genetically encoded biosensor for visualising
hypoxia responses
in vivo. Biol. Open..

[34] Semenza G. L. (2001). HIF-1 and
mechanisms of hypoxia sensing. Curr. Opin Cell
Biol..

[35] Chen H., Zou Y., Shang Y., Lin H., Wang Y., Cai R., Tang X., Zhou J. M. (2008). Firefly Luciferase Complementation
Imaging Assay for Protein-Protein Interactions in Plants. Plant Physiol..

[36] Nooti S., Naylor M., Long T., Groll B., Manu, Hui X. (2023). Manu. LucFlow: A method
to measure Luciferase reporter expression in single cells. PLoS One..

[37] Hung M. E., Leonard J. N. (2015). Stabilization of exosome-targeting
peptides via engineered
glycosylation. J. Biol. Chem..

