# Supplementary material for: Synthetic Phosphorylation Networks with Fluorescence and Luminescence Expansion
Source: ACS Synth Biol. 2025 Jun 6;14(6):2002–11. doi: 10.1021/acssynbio.4c00814 (PMC12186673; doi:10.1021/acssynbio.4c00814)
Supplement: Supplementary file 1 [file sb4c00814_si_001.pdf]

Supporting Information for synthetic phosphorylation networks with fluorescence and luminescence expansion

Leah Davis[+,1], Evan Hutt[+,1], Matthias Recktenwald[1], Samarth Patel[1], Madison Briggs[1], Madeline Dunsmore[1], Sebastián L. Vega[1,2], Mary M. Staehle[1], Peter A. Galie[*,1], and Nichole M. Daringer[*,1]

1. *Department of Biomedical Engineering, Rowan University, Glassboro, NJ, 08028, USA*
2. Department of Orthopaedic Surgery, Cooper Medical School of Rowan University, Camden, NJ, 08103, USA

Corresponding Author: Peter A. Galie, galie@rowan.edu

A

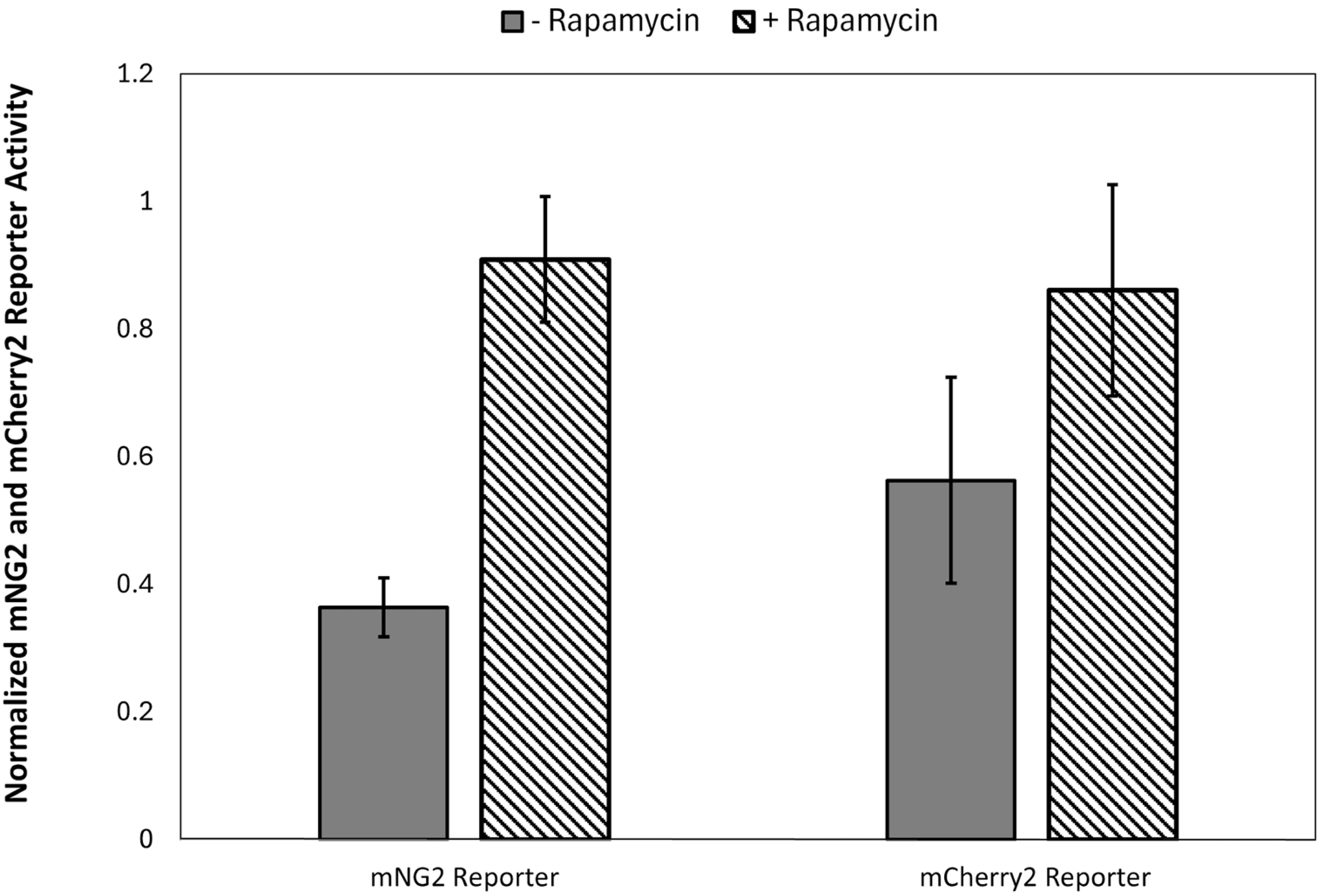

Figure S1 - Bimolecular Fluorescent Complementation (BiFC) comparison of mNeonGreen and mCherry – Normalized mean fluorescence intensity of HEK293 cells transfected with the rapamycin-responsive platform functionalized with either split mNeonGreen or split mCherry and evaluated using flow cytometry. mNeonGreen resulted in a more robust response to rapamycin-induced dimerization when evaluated by flow cytometry and was thus chosen for all subsequent work.

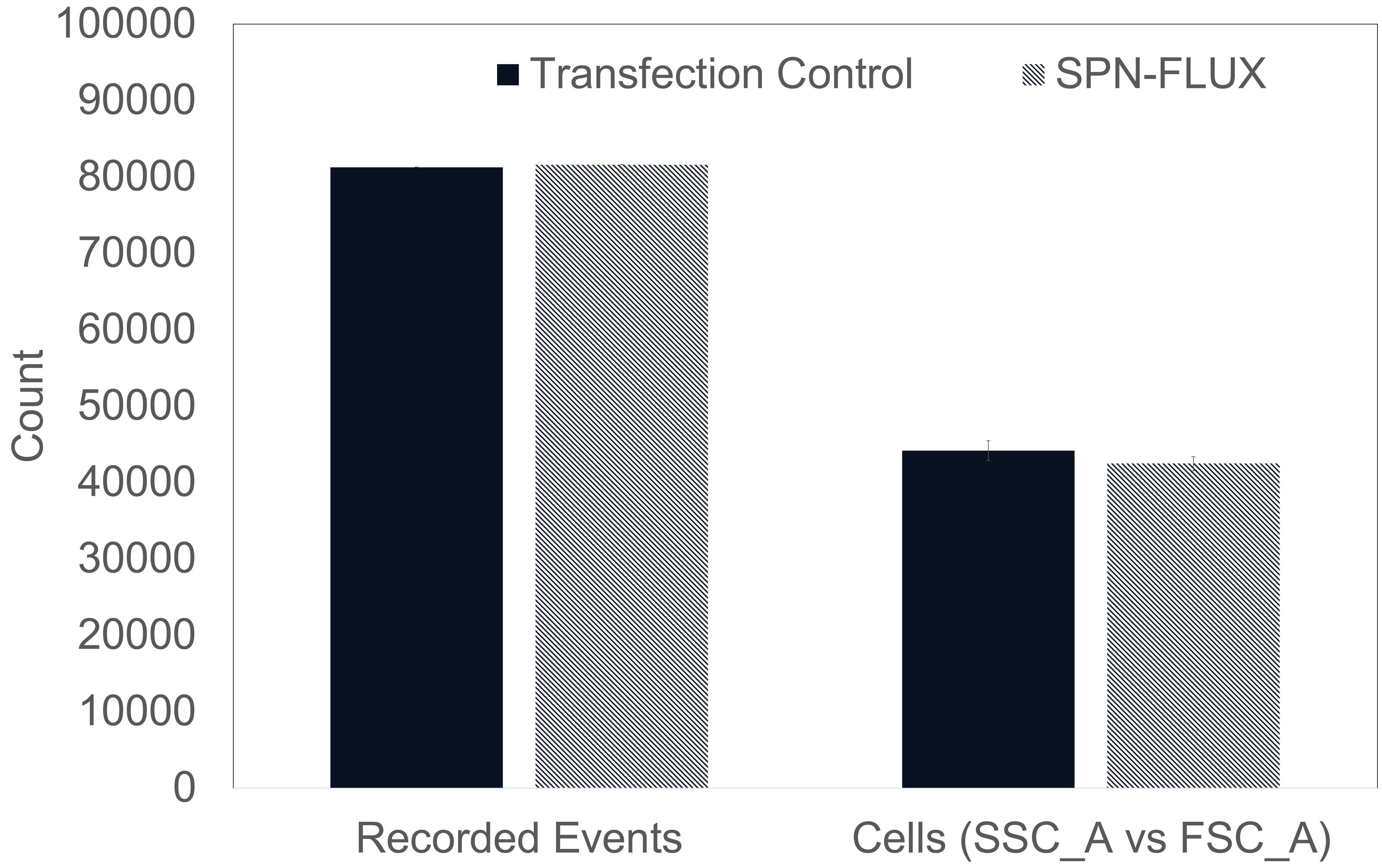

Figure S2 - Recorded events and daughter cell population evaluated by flow cytometry across the transfection control and SPN-Flux platform do not vary, indicating consistent cell growth and supporting lack of platform toxicity.

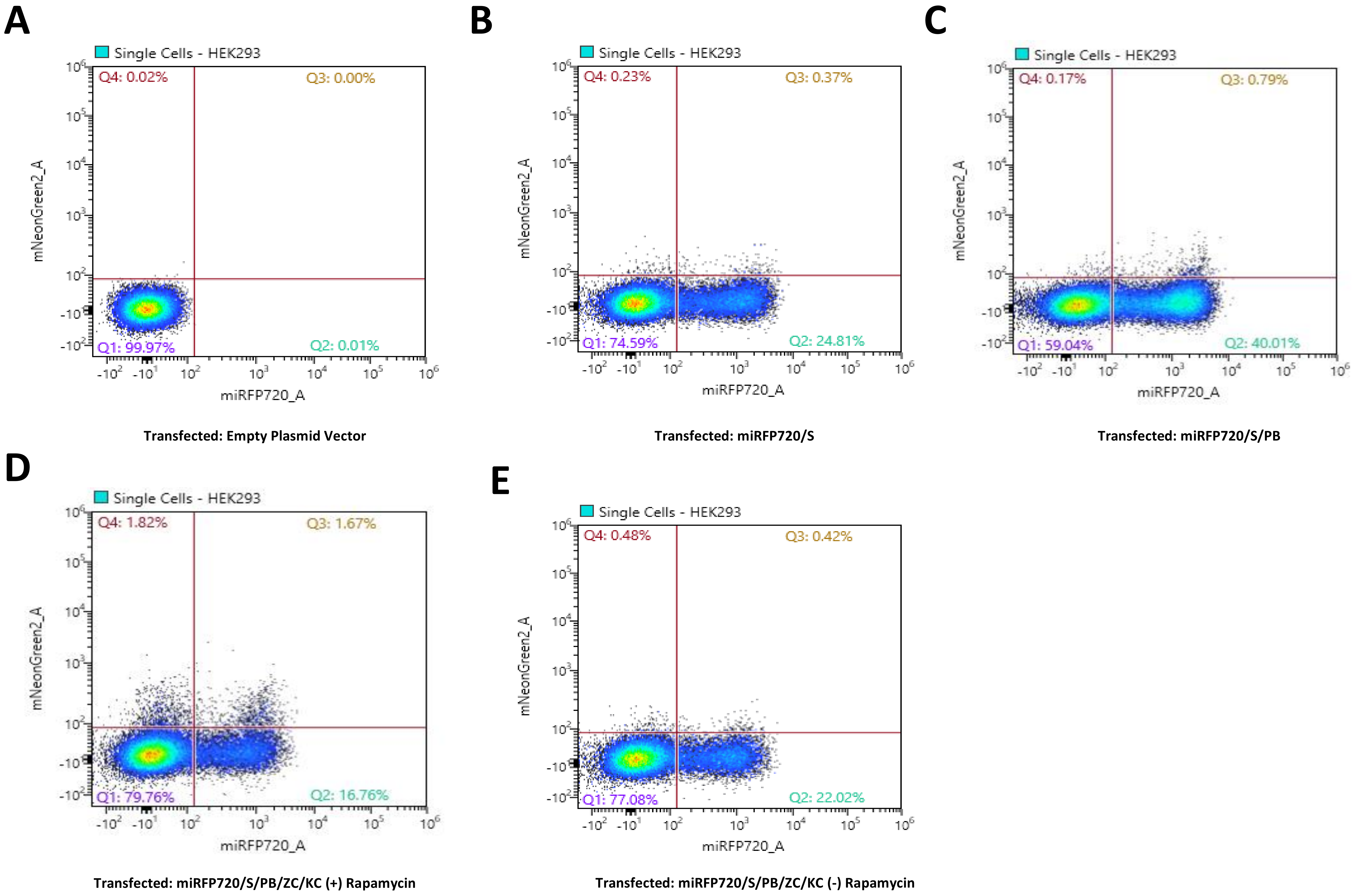

Figure S3 - SPN-Flux transfection groups' single cell population mNeonGreen vs miRFP720 dot plot supports MFI trends in Figure 3. The transfected cell population gate is set by the pc plasmid control in S3A, while the mNG gate is set by the transfected cell population in S3B. S3C represents non-specific BiFC, while S3D and S3E showcase reporting ability.

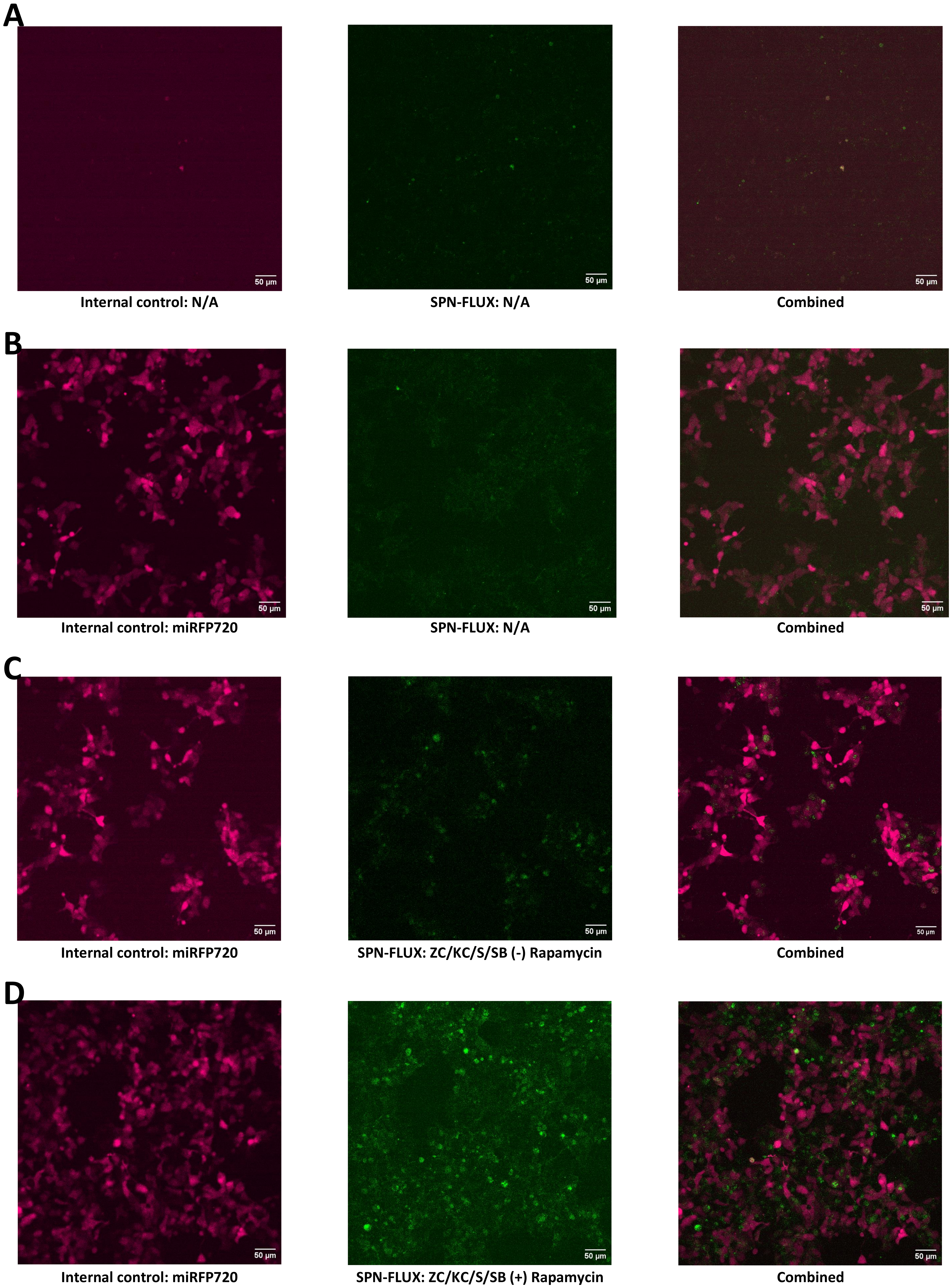

Figure S4 - SPN-Flux in Expressed in HEK293 Evaluated via Fluorescent Microscopy – A) pc plasmid control is negative in both miRFP and mNG channels; B) miRFP Transfection Control shows cells positive for miRFP and negative for mNG; C) SPN-Flux – Ligand highlights the mNG background when the BiFC components are present but unstimulated; D) SPN-Flux + Ligand highlights the reporting ability of the construct.

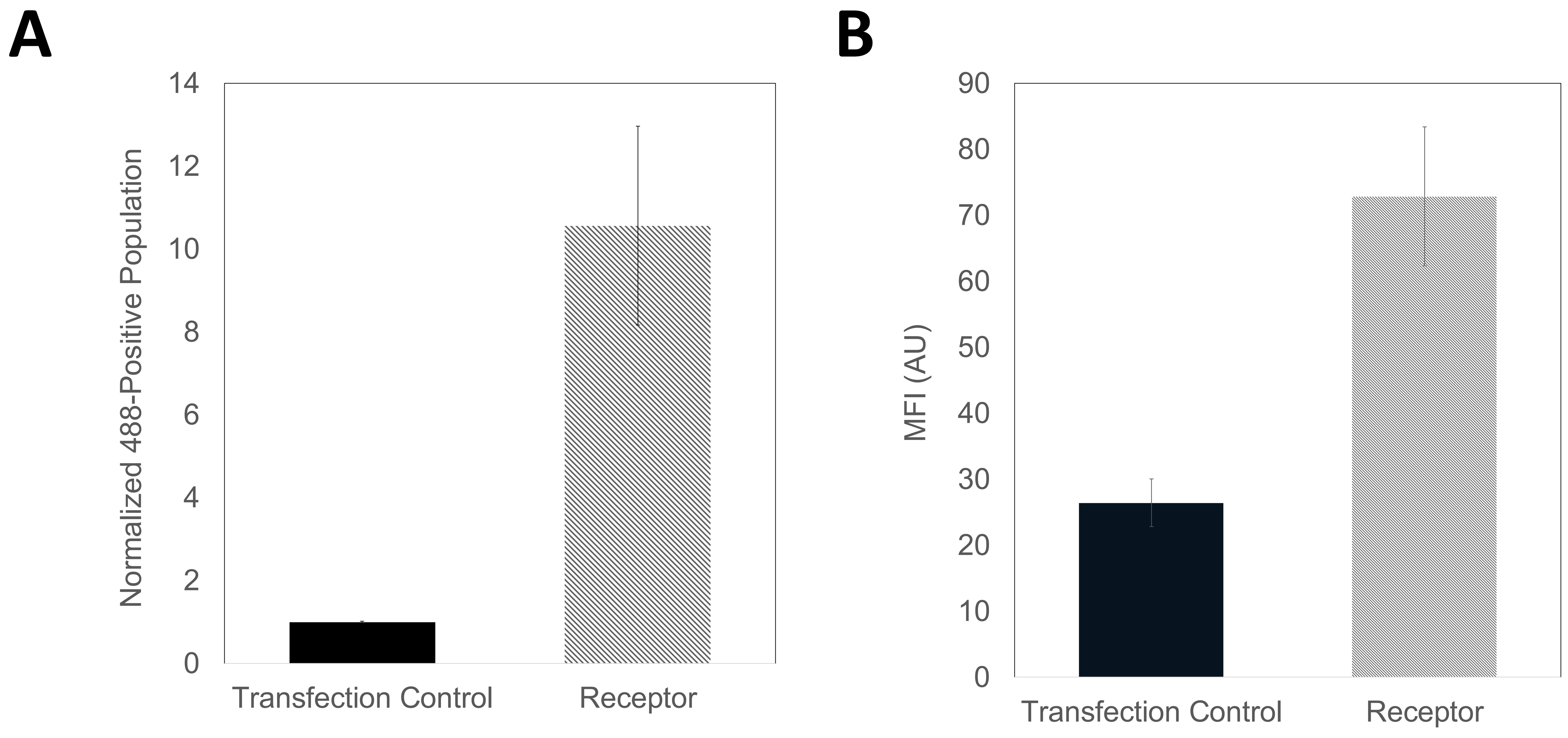

Figure S5 - Synthetic transmembrane receptors are trafficked to the cell membrane, as confirmed by AlexaFluor-488 conjugated HA-antibody treatment of live cells. Significant increases in both (A) the normalized percentage of transfected cells positive for AlexaFluor488 and (B) AlexaFluor488 MFI were observed via flow cytometry, indicative of extracellular synthetic receptor localization.

A

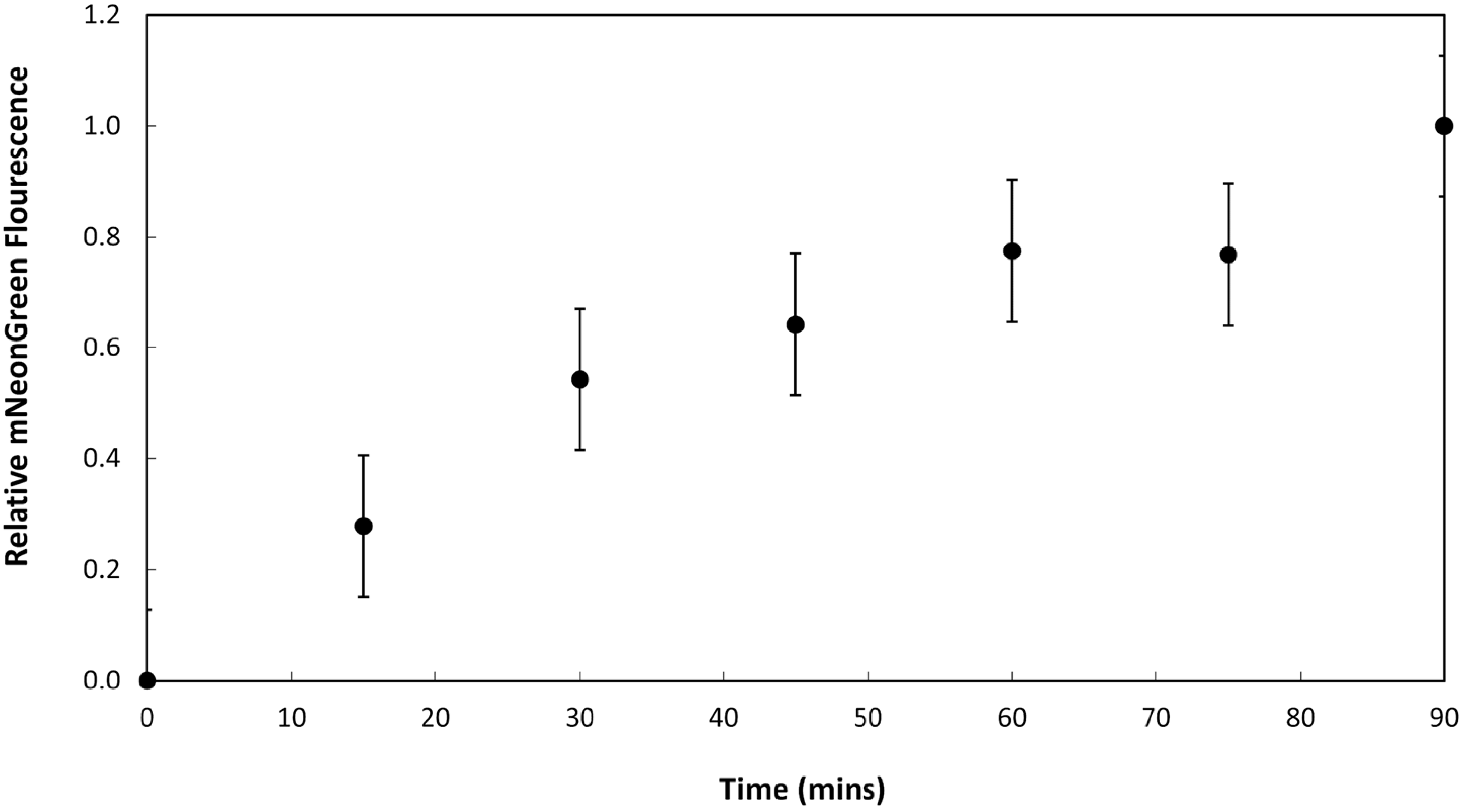

Figure S6 - SPN-Flux activation dynamics are observed over a period of 90 minutes to highlight the post-translational platform's rapid response, evaluated by plate reader. Triplicate samples were dosed at time 0 and evaluated every 15 minutes for 90 minutes.

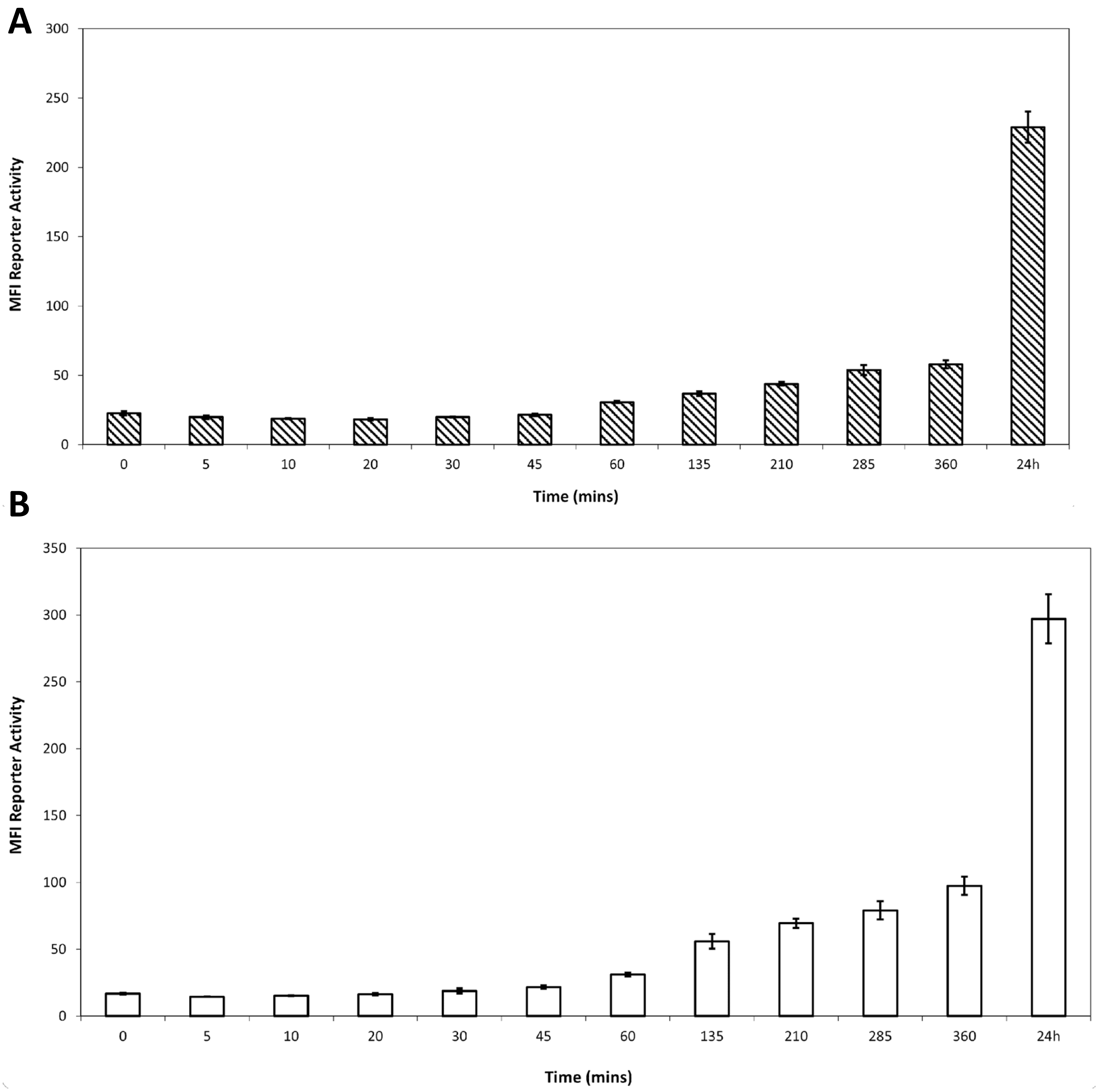

Figure S7 - SPN-Flux activation dynamics are observed over a period of 24 hours for (A) receptor-mediated and (B) constitutively active configurations using a flow cytometer to determine mean fluorescence intensity.

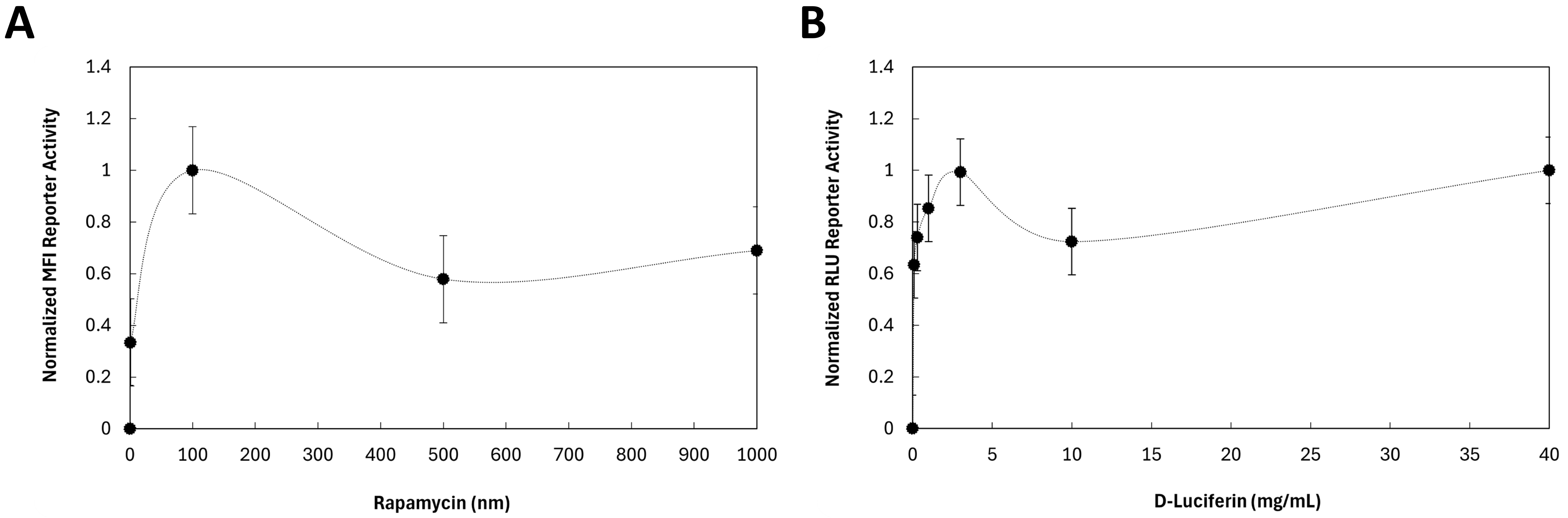

Figure S8 - Rapamycin (A) and D-Luciferin (B) concentration curves evaluated by flow cytometer and plate reader, respectively, indicate target doses for each of the molecules. Doses of 100nM were chosen for Rapamycin and 1mg/mL for D-Luciferin in subsequent experiments.

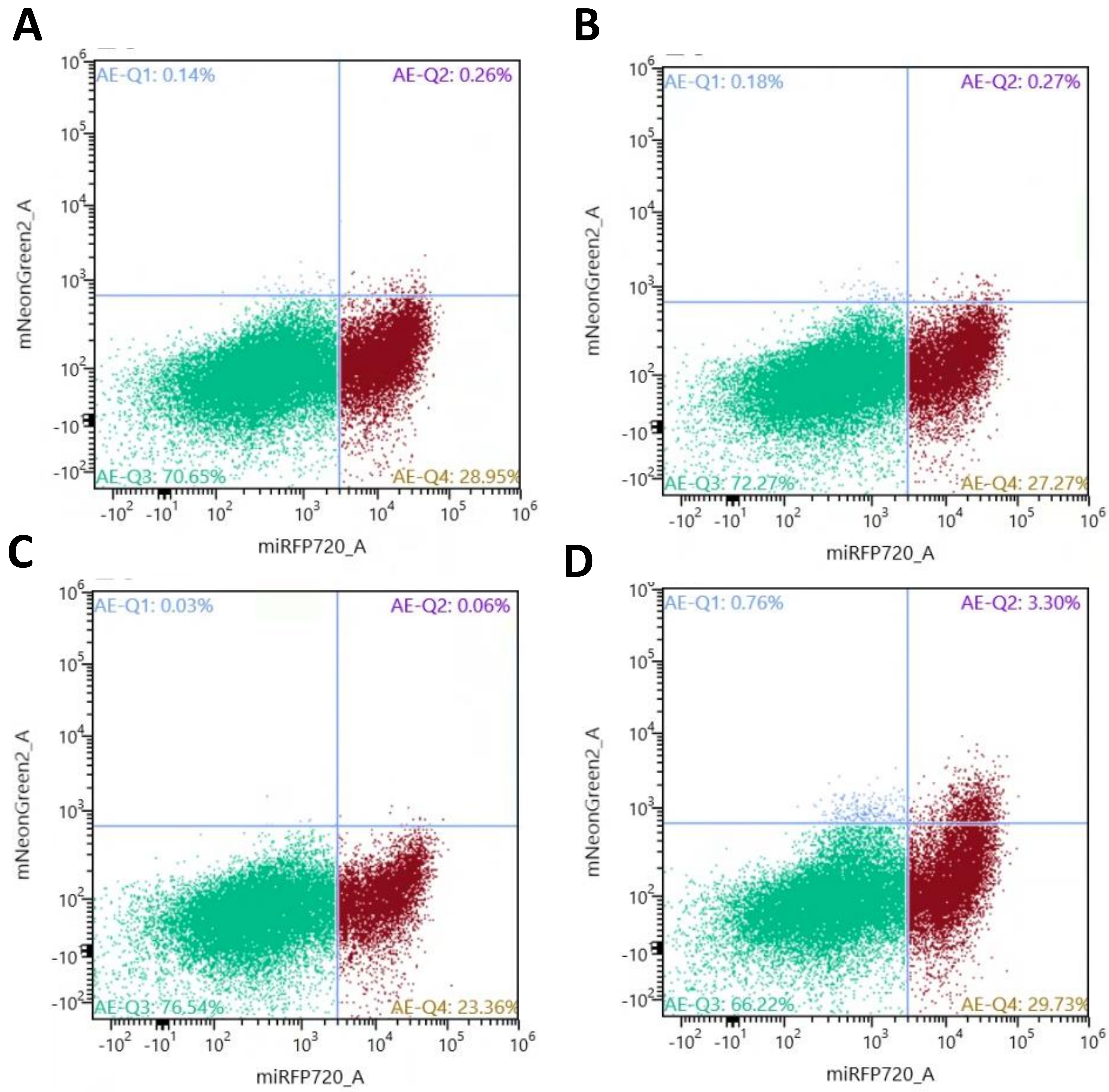

Figure S9 – Representative flow cytometry scatter plots for Figure 5: (A) KZ- ODD- Normoxia, (B) KZ- ODD- Hypoxia, (C) KZ+ ODD+ Normoxia, and (D) KZ+ ODD+ Hypoxia.
